# Punk’s not dead. Fungi for tinder at the Neolithic site of La Draga (NE Iberia)

**DOI:** 10.1371/journal.pone.0195846

**Published:** 2018-04-25

**Authors:** Marian Berihuete-Azorín, Josep Girbal, Raquel Piqué, Antoni Palomo, Xavier Terradas

**Affiliations:** 1 Institute of Botany, Hohenheim University, Stuttgart, Germany; 2 Department of Animal Biology, Plant Biology and Ecology, Universitat Autònoma de Barcelona, Barcelona, Spain; 3 Department of Prehistory, Universitat Autònoma de Barcelona, Barcelona, Spain; 4 Archaeology Museum of Catalonia, Barcelona, Spain; 5 Archeology of Social Dynamics, Institución Milá y Fontanals, Consejo Superior de Investigaciones Científicas (IMF-CSIC), Barcelona, Spain; University at Buffalo - The State University of New York, UNITED STATES

## Abstract

This paper presents the study of the fungi remains preserved in the waterlogged deposits of the Neolithic site of La Draga. These resources had the potential of being used as food and medicine, but also as tinder. Fire was without a doubt one of the most important resources for past people. It was used for lighting, heating, processing food and other materials, cooking and protection, and also possessed social and ritual significance. Hearths are one of the most common features at archaeological sites, but very often little attention is paid to the question of how these fires were lit, and they are seldom reflected in the archaeological record. In order to produce fire by percussion, an intermediate material is required between the sparks and the fuel. Fruiting bodies of fungi are a potential form of tinder, but are less inclined to be well-preserved than other materials. This paper presents the fungal fruiting bodies found at the Neolithic site of La Draga and discusses the meaning of their presence within the archaeological context of the site and European Prehistory.

## Introduction

Beyond their importance for the environment and within the general function of ecosystems, fungi have been important materials for the majority of human societies. Nowadays, they serve a variety of purposes, mainly as food, raw material and medicine. The ethnographic record shows how a multitude of fungi species have been and are still used for a variety of purposes. Their use as tinder in several societies is well known [1] and their importance as food and medicine has been highlighted in the abundant ethnomycological literature [2–4].

Due to their perishable nature, fungi are not very well-preserved in the archaeological record compared to other plant or animal remains [5]. All the archaeological fungi finds to date have been found at waterlogged sites or in permanent ice conditions. However, the scarcity of their remains does not necessarily reflect the importance that they may have had in prehistory [6]. Although the lack of studies on archaeological fungi is significant [7], an increasing number of references to fungi found in archaeological contexts has been published in recent decades. Nevertheless, they often remain unstudied due to the lack of specialists for their identification within archaeological research groups. On the other hand, new methods have recently been implemented to process such finds. In recent studies [8, 9], the presence of fungi have been identified through spores of what could be mushrooms used for food or medicine at the Magdalenian "El Miron Cave", and through DNA at the Neolithic site of La Marmotta (Italy). These finds highlight the necessity of looking for this resource with “alternative” methods. Similarly, the work of O’Regan et al. [10] proposes to demonstrate the consumption of mushrooms by means of stable isotope analysis.

Despite all these difficulties, some fruiting bodies of fungi have been identified in early archaeological contexts, opening a debate on the importance that these resources may have had for prehistoric people. A thorough revision of the recovered remains of fruiting- bodies of fungi is offered by Kreisel and Ansorge [11]. Not every find can be related to human activities, as in the case of the Palaeolithic site of Riesenhirsch in Germany, [12] or the Neolithic La Motte-aux-Magnins in France, where remains of *Fomes fomentarius* were found [1]. In other sites, such as at the Mesolithic Star Carr in England, *Fomes fomentarius* was found in abundance, and possibly used as tinder for fire-lighting [13]. For later chronologies, we have the very popular case of the Iceman, Ötzi, who carried two different fungi species among his travel equipment: *Fomes fomentarius* and *Piptoporus betulinus*, and at least in the case of the first one, it has been interpreted as tinder [14]. In other cases, these remains have not been identified at species level and their use has not been discussed in publications, for instance at the Mesolithic site of Zamostje 2 in Russia, or at the Neolithic of Stare gmajne in Slovenia [6].

This paper presents the fungi assemblage recovered at the Neolithic waterlogged site of La Draga (Banyoles, NE Spain) with the aim of interpreting and discussing their presence. Here, the extraordinary waterlogged conditions of the archaeological units have enabled the conservation of organic material. Understanding the process of incorporation of the fungi to the site is essential to determine the intentionality of their gathering by the inhabitants of La Draga. In order to determine the anthropic origin of these fungi, we combine the ethnomycological data with the analysis of the distribution and physical characteristics of the remains.

### La Draga site

**Fig 1:**
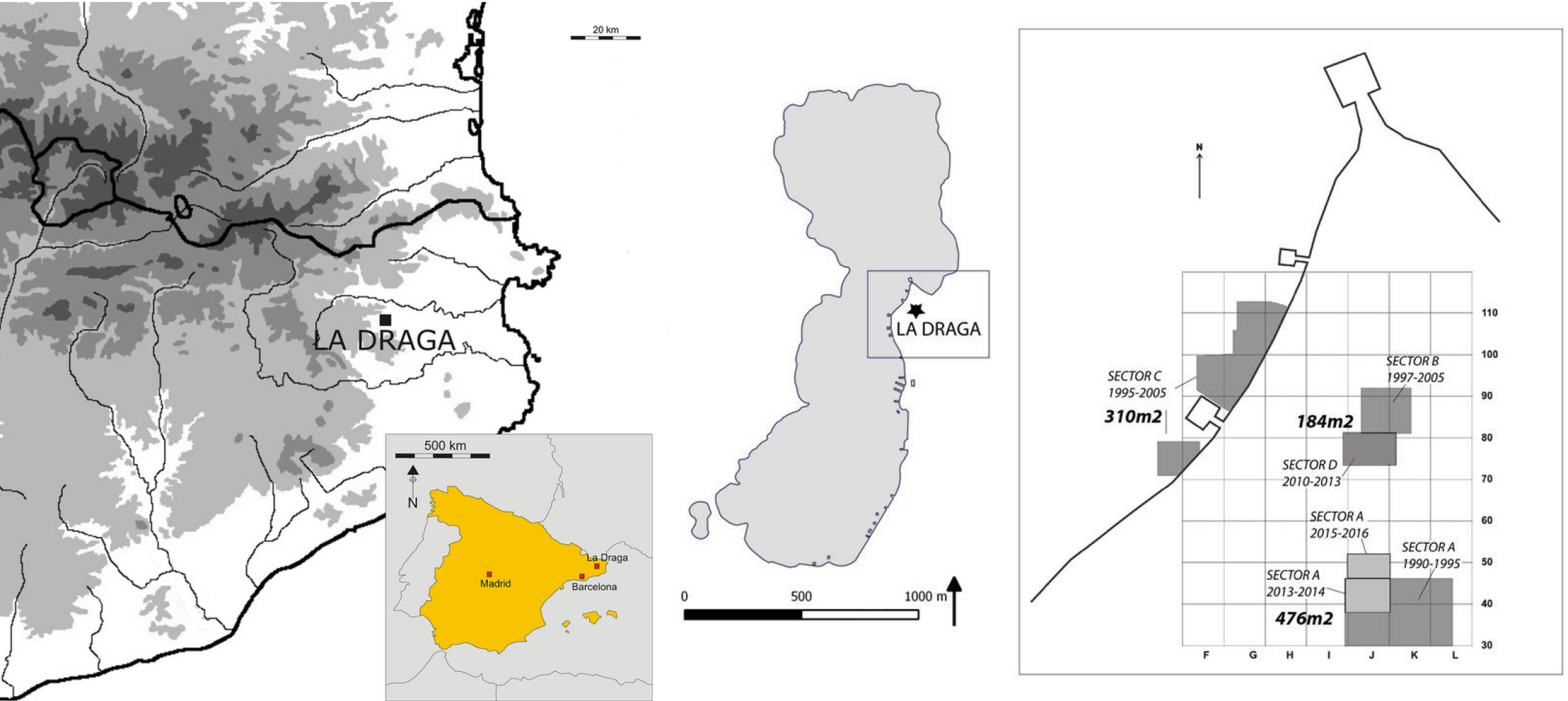
Location of the site.

The Early Neolithic site of La Draga is located on the eastern shore of Lake Banyoles (NE Spain), at 172 m a.s.l. (UTM: 48 01 04 m East and 46 64 097 m Nord), 35 km from the Mediterranean Sea and 50 km south of the Pyrenees (Fig 1). Due to the location of the settlement–on the shore of the lake-, part of the site is currently underwater and the other part along the shore, where archaeological levels are partially covered by the water table. Several sectors have been excavated (Fig 1). Sector A is located in the highest part of the site; in this sector, the archaeological level is above the water table and therefore organic remains have not been preserved, with the exception of the bottom of the posts that have remained under the water table. In Sector B-D, where the samples studied in this article were recovered (see Fig 1), the archaeological layer is below the water table. Finally, Sector C corresponds to the underwater part of the site and, although the organic material is well preserved in this sector, no remains of fungi have been recovered.

The site corresponds to an open-air settlement whose surface area is estimated to exceed 15,000 m^2^, of which 959 m^2^ have been excavated since 1991 [15–17]. The most recent work undertaken since 2010 has shed light on the stratigraphy, the chronology and the characteristics of the occupations.

Two phases of occupation with distinctive construction traditions have been documented, both attributable to the late Cardial Ware Neolithic culture according to the ceramic assemblage [18,19]. The radiocarbon dates available so far allow us to date the occupation of the settlement in the late sixth millennium and early fifth millennium cal BC [20,21]:

- Phase I (5324–4977 cal BC) is the oldest occupation. The use of wood to build dwellings and structures is documented in the layer formed by the collapsed wooden constructions. In this layer, organic materials are very well preserved due to their position below the water table.
- Phase II (5210–4796 cal BC), is characterized by the use of travertine slabs for construction. The layer that corresponds to this phase is currently below the water table although at some time remained above it, for this reason, within this layer, organic material is only preserved by carbonization.

The overlapping travertine structures document two different construction phases but radiocarbon dating does not reveal a chronological hiatus that might indicate a period of abandonment of the settlement. The two occupations were very close in the time; moreover, subsistence activities as well as technical features and technological traditions attested by means of artefact analysis indicate continuity over time [18,20].

The reconstruction of subsistence activities indicates a well-established farming economy mainly based on livestock and cultivation, as shown by archaeozoological and archaeobotanical evidence. Among the cereals, naked wheat was the main crop (*Triticum aestivum*, *T*. *durum*, *T*. *turgidum*), although barley and naked barley (*Hordeum vulgare* and *Hordeum vulgare var*. *nudum*) were also cultivated, along with emmer (*Triticum dicoccum*), einkorn (*T*. *monococcum*), peas (*Pisum sativum*) and poppy (*Papaver somniferum*, *P*. *setigerum*) [22]. As regards livestock, ovicaprids, pigs and cattle constituted the main species [23]. Despite the important role of domestic plants and animals in their subsistence, the inhabitants of La Draga exploited a wide range of wild animal and plant resources, for both technological and food purposes. To this group of gathered staples belong fungi, the object of the present study. In the oldest phase of occupation, the preservation of wooden implements, such as digging sticks, sickles and bows, as well as fibres, provides extraordinary insights into woodworking, cordage production and the techniques of food procurement, especially in relation with agricultural and hunting activities [24–28].

## Materials and methods

The fungal remains recovered at La Draga come from the oldest occupation phase at the site and specifically from Sector B-D (144 m^2^ excavated from 1997 to 2005 and 58 m^2^ between 2010 and 2013) (see Fig 1). All fungal remains have been preserved thanks to waterlogged conditions. They were recovered during the field excavation and subsequent water screening of the sediments. Afterwards, the items were preserved in water and cold storage. Currently, 86 remains of fungi, complete and/or fragmented, from 46 different squares of the excavation grid, constitute the totality of the assemblage. An initial identification of the remains from the excavations undertaken between 1990 and 2004 has been published [29,30]. These papers, where only 45 of the remains were taken into account, provide exclusively a list of the specimens and their taxonomical adscription. In contrast, the present paper includes all the remains recovered to date: the 45 previously published, 24 recovered during the 2010–2012 field seasons and 17 from old excavations. All have been studied to determine the species. In addition, the specimens have been measured, whenever possible, and such other features as their general state of preservation or the evidence of manipulation have been recorded (S1 Table). Finally, the taxa distribution in the site has been analysed.

The remains were identified in the Department of Biology at the Autonomous University of Barcelona, according to anatomical morphometric and biometric characteristics. Parameters offered by specific literature were followed [31–33].

The structure of the fruiting body hyphae, the number of pores per unit area and the consistency of the characteristics of the surface are considered to identify the species. The remains have been submerged, which leads to darkening of the structures and the loss of some distinctive elements, hindering the comparison with individuals in a natural state and with modern literature to achieve identification. In some cases, the state of degradation has limited identification to genus level.

## Results

A total of 84 out of 86 remains have been identified (Table 1). The six taxa represented are: *Skeletocutis nivea*, *Coriolopsis gallica*, *Daedalea quercina*, *Daldinia concentrica*, *Ganoderma adspersum*, and *Lenzites warnieri*.

**Table 1:** Identified fungal remains by species.

Species	Common name	Number
***Coriolopsis gallica***	Brownflesh bracket	8
***Daedalea quercina***	Oak mazegill	9
***Daldinia concentrica***	Cramp Balls	11
***Ganoderma adspersum***	Southern Bracket	51
***Lenzites warnieri***	-	2
***Skeletocutis nivea***	Hazel bracket	3
**Unidentified**		2
**Total**		86

Currently the identified fungi can be found in different ecosystems and possess a variety of known uses.

**Fig 2:**
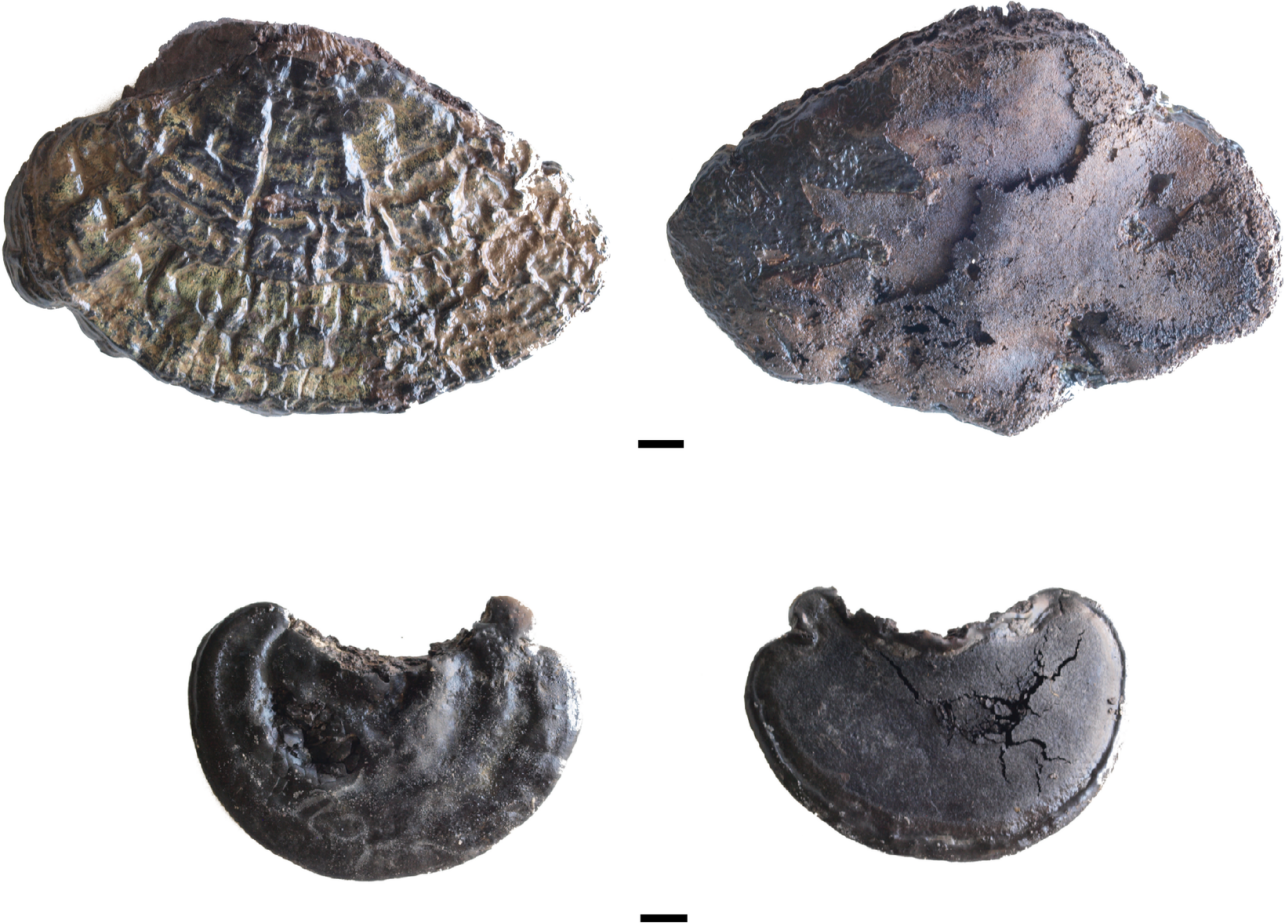
Fruiting bodies of *Ganoderma adspersum*.

**Fig 3:**
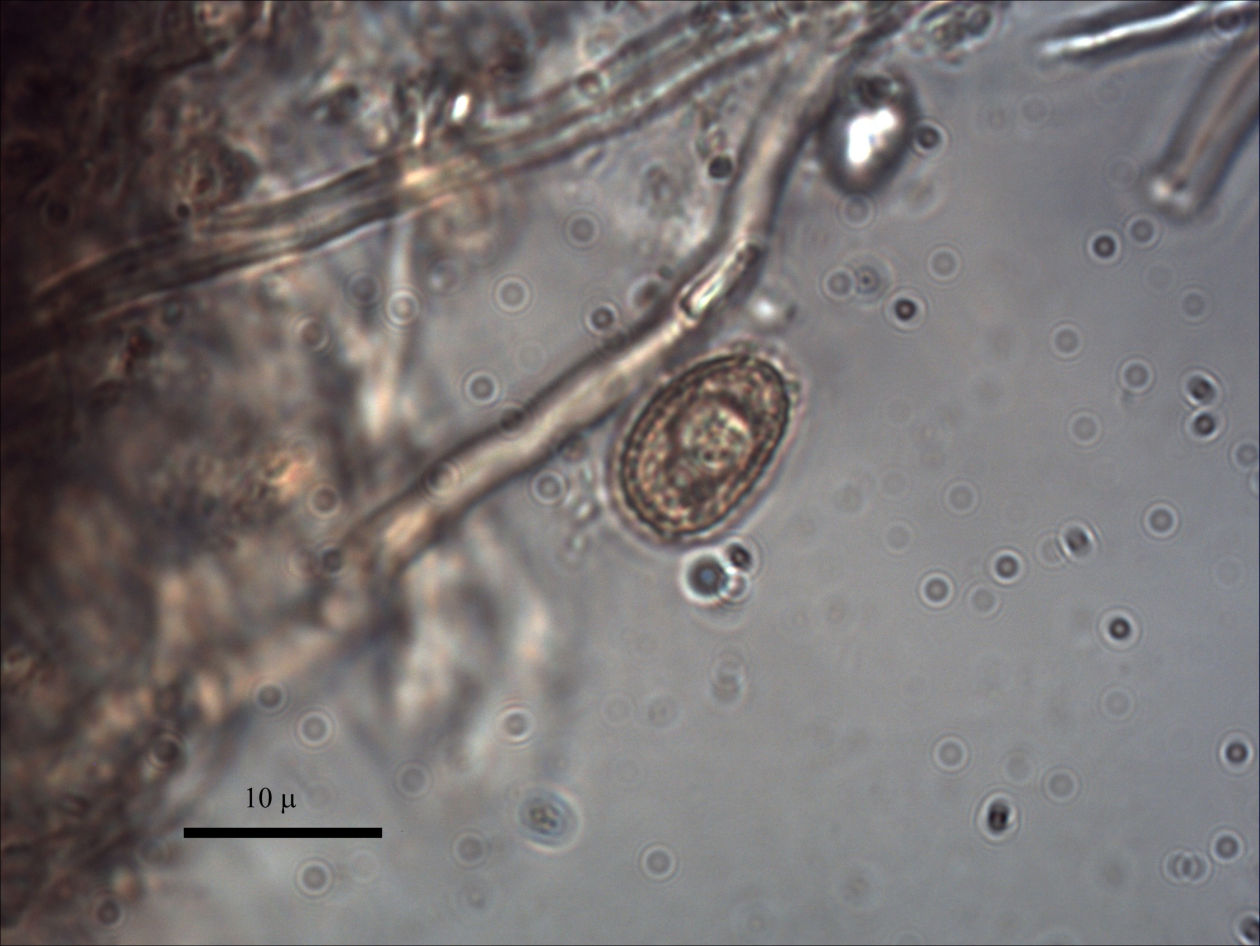
Spores of archaeological *Ganoderma adspersum*.

***Ganoderma adspersum*** (Schulzer) Donk (Fig 2). The fruiting body of the southern bracket is 7–60 cm long, 5–25 cm wide and 3–30 cm thick; the upper surface has a thick dark brown, hard knobbly crust which is concentrically ridged, and the margin is thick and obtuse. The number of pores on the lower surface is 3–4 per mm^2^. Typical *G*. *adspersum* spores were observed with a microscope. They are ellipsoid and the distinguishing feature is that the spore is double-walled with a darker inner layer carrying a particular cell pattern that pierces the outer hyaline, so that the spore seems to have a spiny surface (see Fig 3) [34]. This polypore is a parasitic fungus of multiple species, both evergreen and deciduous. The fructification is annual any time of the year; therefore, it is available all year round. The fruiting bodies of *G*. *adspersum* grow mostly on the stem base of hardwoods; its hosts include the genera *Tilia* and *Quercus*. This fungi has good properties as tinder, and once it is ground it takes fire easily [1]. In the case of La Draga, *G*. *adspersum* is the main taxon, with 51 out of 86 identified remains.

**Fig 4:**
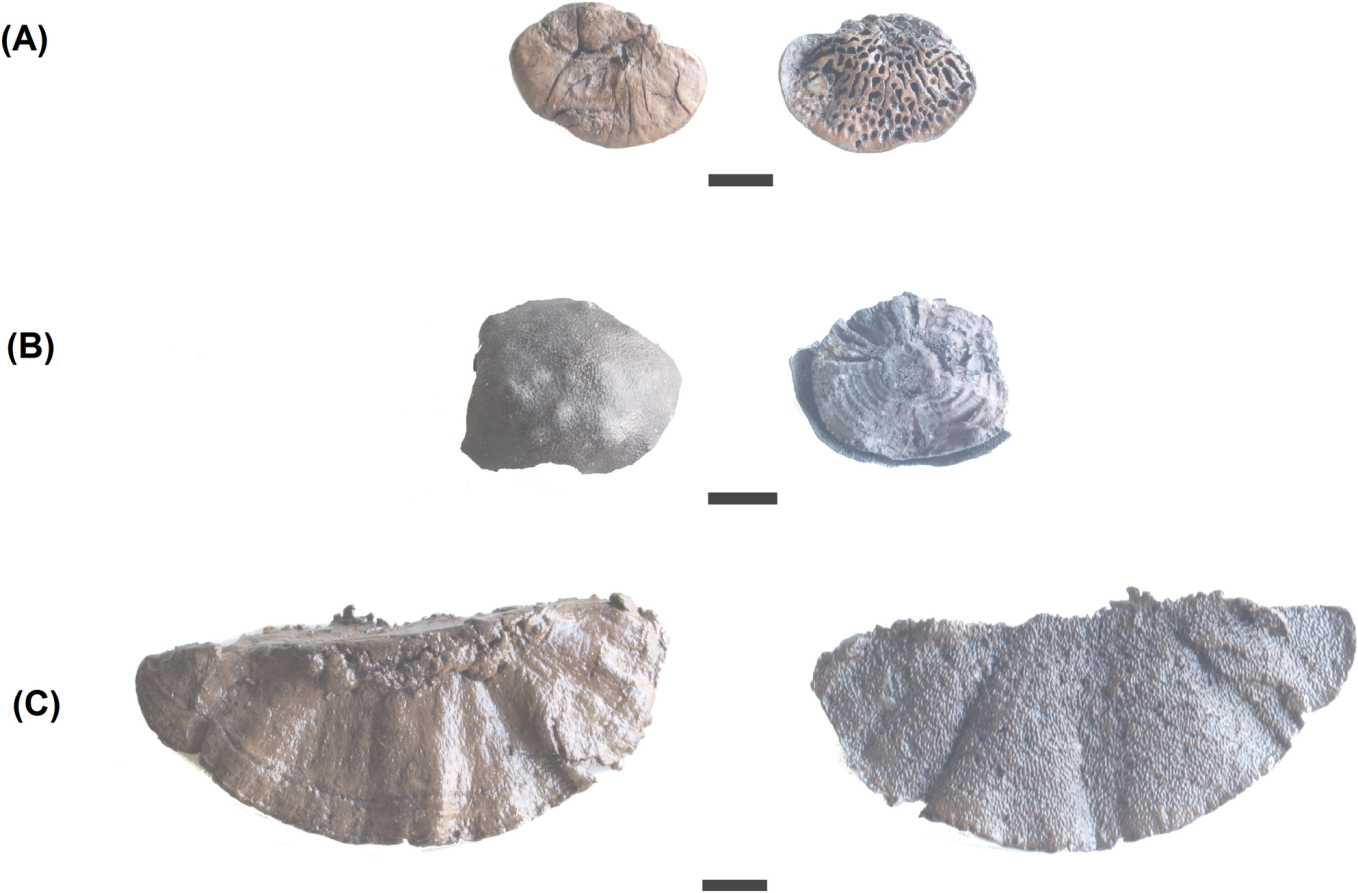
Examples of fruiting bodies recovered at La Draga site. (A) *Daedalea quercina*; (B) *Daldinia concentrica*; (C) *Coriolopsis gallica*.

***Daedalea quercina*** (L.) Pers (Fig 4A). The fruiting body of the oak mazegill is solitary or imbricate, forming roughly semicircular brackets 5–20 cm long, 6–10 cm wide and 2–5 cm thick. The underside surface has mostly labyrinthine pores with some of the walls resembling forked gills. It frequently grows on dead oak wood, and is annual or perennial, in the latter case available all year round. If its surface is scraped, it takes fire easily and beating and stretching render it more effective, as in the case of *Fomes fomentarius* [1]. Besides its use as tinder, other uses are known ethnographically: as a comb, for flavouring and as a haemostatic [1].

**Fig 5:**
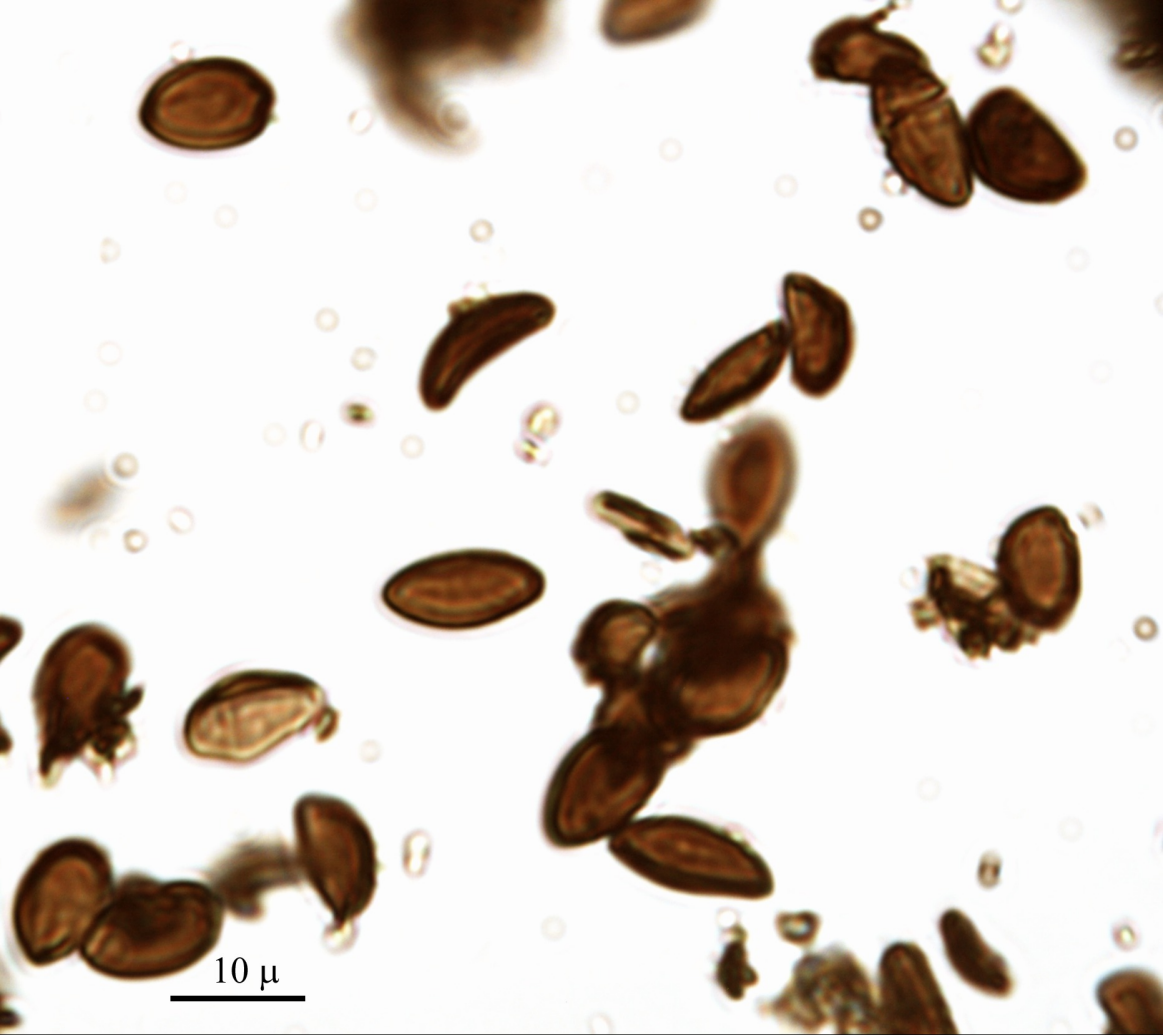
Spores of archaeological *Daldinia concentrica*.

***Daldinia concentrica*** (Bolton) Ces. & De (Fig 4B). The stromata called cramp balls or King Arthur’s Cakes [35] are hemispherical, up to 4 cm in diameter. They reveal characteristic pale and dark concentric zones when cut vertically through the middle. Typical *Daldinia concentrica* spores were observed with a microscope. They are ellipsoidal to fusiform, 12–17 x 6–9 μm (Fig 5). They grow on decaying or dead wood of deciduous trees, especially on trees of the *Fraxinus and Quercus* genera. The fructifications are perennial and can be observed at any time of the year. This species is a common source of tinder as, once dried, it ignites very easily, although without an open flame. For this reason, it is quite useful to transport fire when moving from place to place.

***Coriolopsis gallica*** (Fr.) Ryvarden (Fig 4C). The fruiting bodies of brownflesh brackets are semicircular brackets, 2–15 cm long, 1–7 cm wide and 0.5–1 cm thick. The topside is somewhat zonate and radially furrowed. The underside surface has angular pores 1–3 mm in diameter. They grow on dead or decaying wood of different species, among them *Quercus*, *Fraxinus* and *Ulmus* and are available all year round. Their fleshy part takes fire quickly when ground [1].

***Skeletocutis nivea*** (Jungh) Jean Keller. The fruiting body of hazel bracket is usually 2–6 cm long, 1–3 cm wide and 2–4 mm thick. The underside surface is covered in very small pores, 6–8 per mm^2^. It can be observed at any time of the year. It grows on dead branches of hazel or ash. Three of the recovered individuals correspond most likely to this species [29].

***Lenzites warnieri*** Durieu & Mont. The fruiting body of *Lenzites* is a semicircular bracket with a typical lamelliform-labyrinthian fruit layer, with large-sized gills (the only part identified in La Draga). This polypore grows on the decaying trunk of deciduous trees near the water in regions with a Mediterranean and humid climate. The hosts are usually willow (*Salix* spp.), elm (*Ulmus* spp.), cottonwood (*Populus* spp.), alder (*Alnus* spp.) and other species found in warm, moist areas. Its fructifications are perennial and can be observed at any time of the year, although their greatest development is between September and November. After scraping its surface to oxygenate the fibres, it lights quickly [1]. It is not the most effective tinder, but could be used to light a fire if necessary.

**Fig 6:**
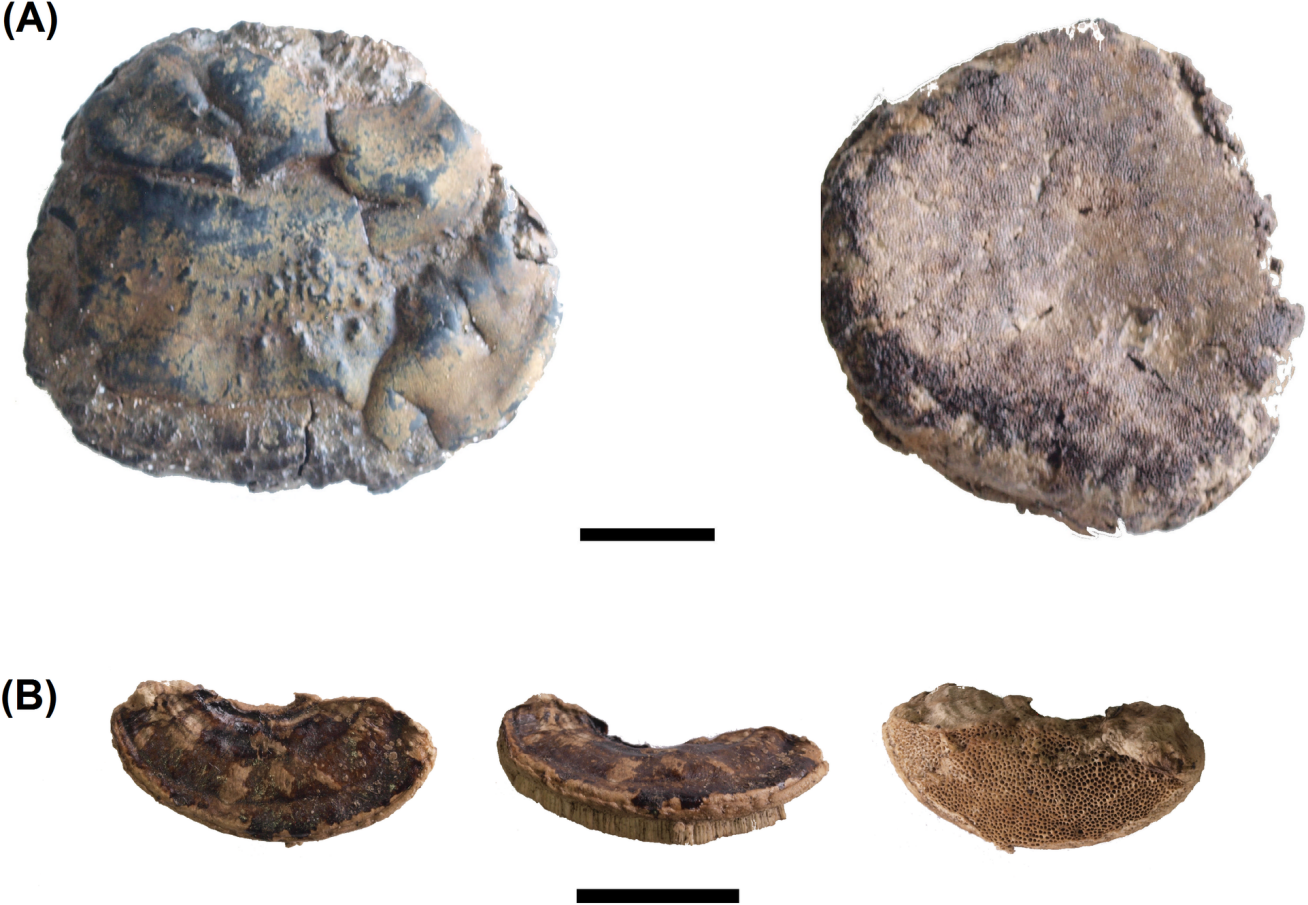
*Ganoderma adspersum* remains with evidence of manipulation.

**Fig 7:**
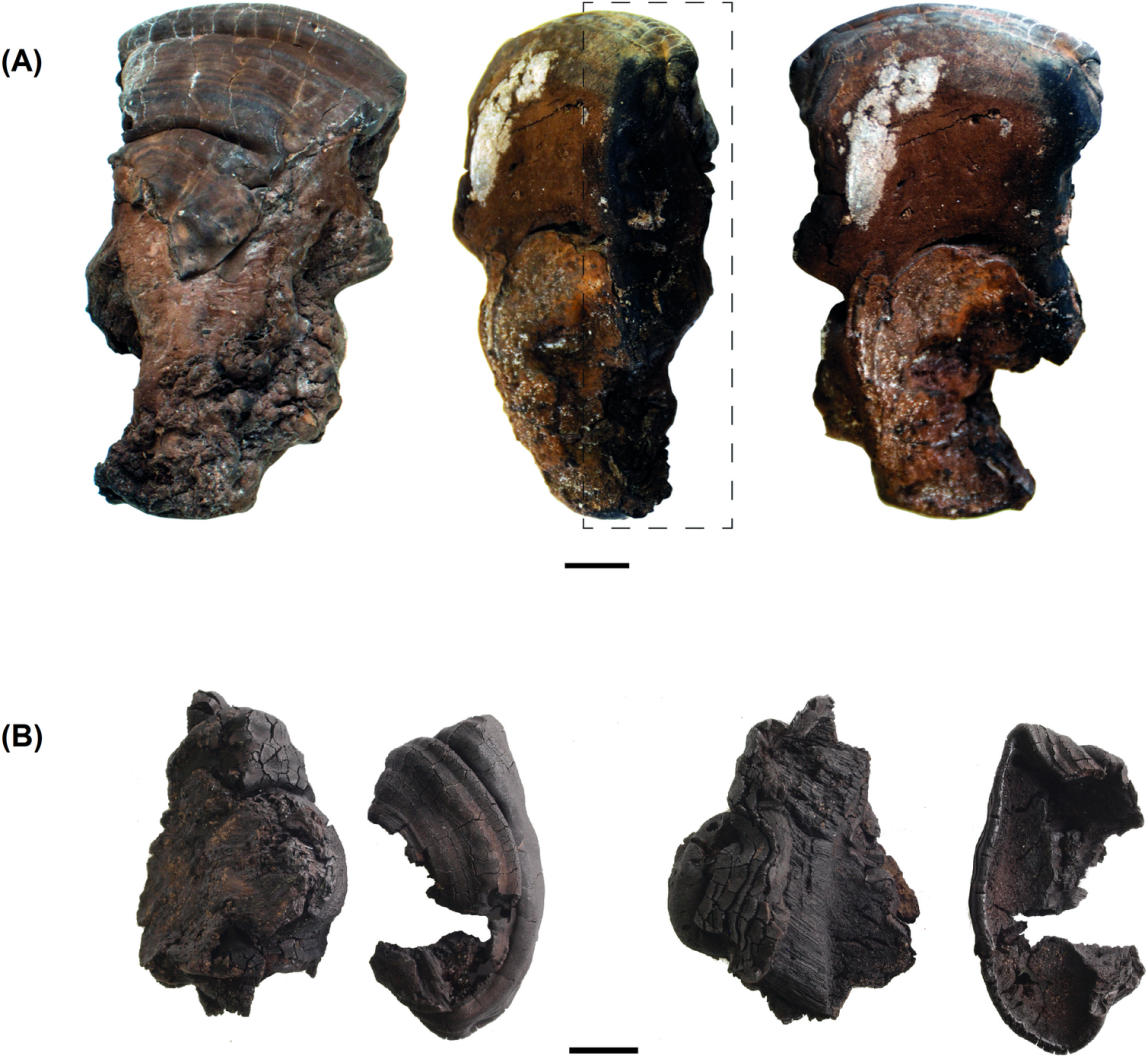
*Ganoderma adspersum* remains partially carbonized. In (A) the dashed box indicates the charred region of the fruiting body.

**Fig 8:**
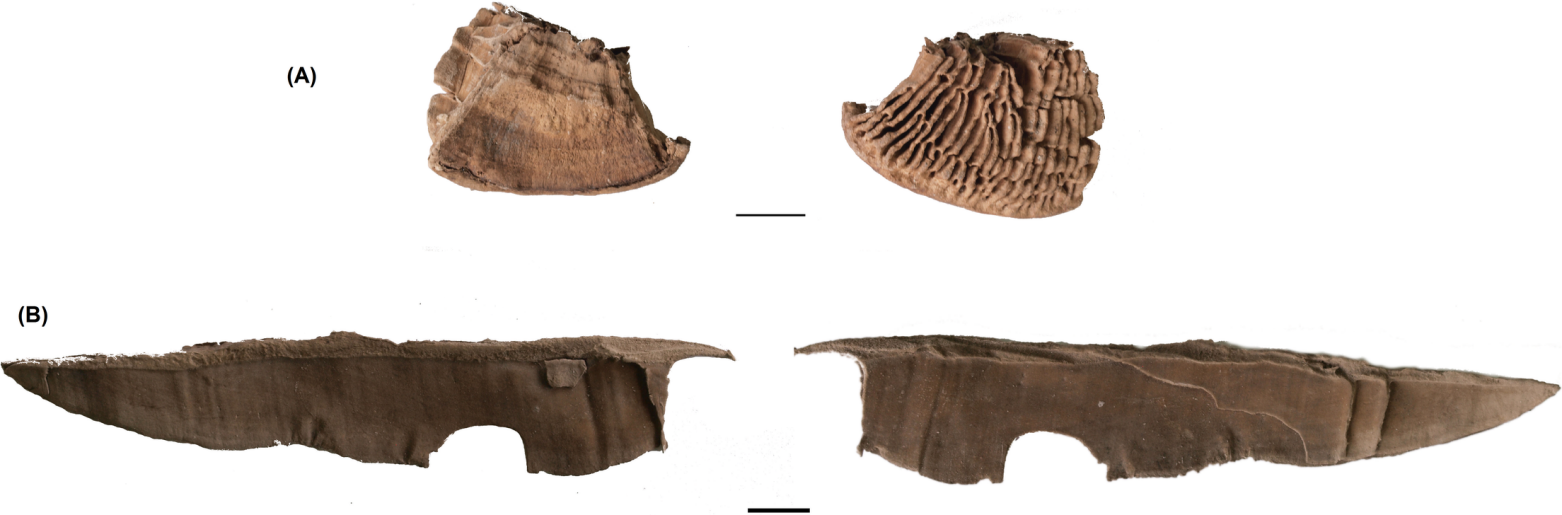
Lenzites warnieri. (A) Fragment and (B) lamella.

Some of the fungi are fragmented; however, 50 individuals are complete. The size of the whole fungi ranges between 10x9x12 mm and 98x140x29 mm, the majority being less than 48 mm long and 62 mm wide. Two individuals of *Ganoderma adspersum*. show signs of manipulation; in these cases, the entire perimeter has been cut (Fig 6), while another two specimens of *G*. *adspersum* and one unidentified individual show evidence of carbonization (Fig 7). According to their sizes, two of the manipulated fungi can be classified among those of small-medium dimensions, with sizes of 18x8x3 and 40x35x 14 mm, respectively. Those partially carbonized ranges between 24x20x10 and 47x86x24 mm. Finally, the *Lenzites warnieri* specimen corresponds to a fragment of a lamella 120x23x3 mm in size that seems to have been prepared intentionally (Fig 8B). Specific information about the specimens studied, their measures and state of preservation is presented in the supplementary material.

**Fig 9:**
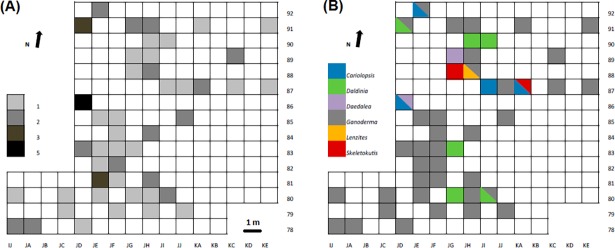
Spatial distribution of fungi remains at the site. (A) Shows concentration of remains and (B) species distribution.

Regarding the spatial distribution of the fungal remains, in Sectors B-D fungi are dispersed across the sectors. In most cases, there are only one or two fungi per grid square. However, in three squares the concentration is higher: in JE-81 and JD-91 with three specimens and JD 86 with five (Fig 9).

## Discussion

### Ecology and origin of La Draga fungi

All the taxa identified correspond to polypore mushrooms. According to their current hosts, all of the fungi identified at La Draga could have grown in the woods near the settlement. The charcoal and pollen data at the site have provided a detailed picture of the vegetation in the early Neolithic [36–39]. The settlement was located on a carbonated sand beach on the eastern bank of the Banyoles Lake. This space was surrounded by a riparian forest consisting of *Laurus nobilis*, *Sambucus* sp., *Salix* sp., *Populus* sp., *Alnus* sp., *Fraxinus* sp. and *Corylus avellana*. In this riparian forest, *Coriolopsis gallica*, *Skeletocutis nivea*, *Daldinia concentrica* and *Lenzites warnieri* would have found the hosts and the moist conditions they needed to develop. Deciduous forest is also well documented in the area, since both charcoal and pollen data have provided evidence of a dense oak forest near the settlement. In these woods, *Daedalea quercina* and *Ganoderma adspersum* may have found an ideal place to grow.

Some of the polypores documented at the site grow on dead wood, which is the case for *Daldinia concentrica*, *Skeletocutis nivea*, *Coriolopsis gallica* and *Daedalea quercina*. In contrast, *Ganoderma adspersum* and *Lenzites warnieri* parasite living trees, inducing their death. The main question is if the fungi were brought to the site consciously: intentionally gathered and transported to be used; or if they are the result of other activities, for example, the transport of dead wood to the site to be used as firewood. Additionally, it is also possible that at least some of the fungi could have grown on the wooden pillars and beams of the dwelling structures during the time that they were in use or after abandonment.

The *in situ* growth of the fungi can be ruled out, at least in the case of *Ganoderma adspersum* and *Lenzites warnieri*. As mentioned above, both are parasites of living trees, which suggests that these fungi could not grow on the poles of the dwellings. Concerning the other identified fungi, their ecological requirements and hosts are diverse, and, for this reason, they were most likely not growing at the same place. The proximity of a riparian forest may have favoured such as fungi as *Skeletocutis nivea*, *Daldinia concentrica*, or *Coriolopsis gallica*, which grow in those environments, reaching the settlement. However, they have been documented in the same places as *Ganoderma adspersum*, which suggests that they have a similar origin.

The unintentional transport to the settlement together with firewood or other wood cannot explain the presence of all the fungi recovered at the site. Wood affected by parasites was probably avoided as building material due to its fragility. In the case of *G*. *adspersum* for instance, an intensive white rot is produced, causing the death of the host and the fracture of the main trunk at the point of decay. The possibility that some of the fungi might have reached the site together with the gathered wood cannot be excluded, since one of the *G*. *adspersum* individuals was still attached to a pole. However, it is the sole case among all the recovered fungi that were found in the same archaeological level.

We believe that most of the fungi recovered at La Draga were transported to the site with the intention to be used. Several factors support this hypothesis. First of all, the ecology of the taxa indicates that these fungi did not grow in the same type of forest; they come from at least two different ecosystems: riparian and oak woods. Both types of forests are well-documented in the area, but the place where dwellings were built was a seasonally flooded lacustrine beach, an open space without arboreal vegetation. In this environment, fungi would not find a suitable host to grow on.

The second reason is related to the abundance of fungi recovered at the site. The 74 individuals were found in a relatively reduced space within Sectors B-D. Although they are distributed over a surface of 202 square metres, there are two main areas of concentration: one in the north and one in the south (Fig 9). Both areas are related to the collapse of dwellings, and abundant wooden remains as well as other artefacts have been recovered. In addition, taxa from diverse ecological origins coexist within this reduced space, which reinforces the hypothesis of an intentional transport to the site.

Finally, the majority of the fungi of La Draga have in common their historical use as tinder and, at least in two cases, display evidence of manipulation. The range of sizes represented is clearly biased towards medium and small sizes, which suggests an anthropogenic selection of individuals (S1 Table). It should be noted that some of the fungi recovered at La Draga correspond to young or very small specimens, and they are rarely larger than 48 x 62 mm, while normally *Ganoderma adspersum* can easily reach diameters of up to 30–40 cm.

### Potential uses of fungi of La Draga: Ethnomycological and ethnographic evidences

Some of the polypores identified at La Draga have been reported at other Neolithic sites in Europe. *Daedalea querciania*, for instance, has been reported at the Neolithic sites of Ergolzwil 4 (Switzerland) and Haithabu (Germany), while *Lenzites warnieri* and specimens of *Ganoderma* have been identified at the Neolithic site of Burgäschisee Süd (Switzerland).

The use of fungi as tinder has been documented for many human groups in historical times. In the northern hemisphere, one of the most popular species used as tinder is *Fomes fomentarius* [40,41]. It is highly flammable and was used to start or transport fire. Its use as tinder has also been documented for prehistoric times, like the remains found among the equipment that Ötzi was carrying with him [42] which were clearly dried with this finality. *F*. *fomentarius* has also been documented at several archaeological sites, with chronologies ranging from the Mesolithic to the Bronze Age [1]. We can mention its presence in Starr Carr [13], Gisement “des Baigneurs” (Charavines, France), or Zurich-Alpenquai (Zurich, Switzerland), among others [1].

Other polypore species have played a similar role. Among the most common ones are *Daedalea quercina* and *Ganoderma lucidum* [1], while this use has been also reported for *Ganoderma*, *Coriolopsis gallica*, *Daldinia concentrica*, that are also grouped under the category of tinder fungi. All these fungi share a series of characteristics such as their woody structure and flammable capacity, which make them excellent materials for punk.

Of the six taxa documented at La Draga, five are known tinder fungi and only *Skeletocutis nivea* has no known uses. It is worth mentioning that only three remains of this taxon have been documented and that it typically proliferates on *Corylus avellana*, a species growing around the site. Those taxa are not edible and although some of them have known medicinal properties, as is the case of *Ganoderma adspersum*, *Daedalea quercina* and *Cariolopsis gallica* [43–45], we believe that the main use of these species at La Draga was as tinder.

Before chemical or modern fire-starters were developed, two methods were used. One consists of striking a piece of flint against a stone rich in ferric sulphur, such as pyrite or marcasite, while the other is based on friction and the heat generated by rubbing together two pieces of wood (with a variety of forms and possibilities). Both methods are attested archaeologically and well known by ethnography; however archaeologists are not able to say which one is older [46]. Regarding the European Neolithic, both methods are known. Boards and drills are known from some Swiss Neolithic lake shore sites [46].

Additionally, evidence of ferric sulphur has been recorded at several Neolithic sites [1]. At La Draga, no evidence has been found of either the use of board and drills or ferric sulphur. However, to light a fire using percussion it is absolutely necessary to have a material that acts as an intermediary between the sparks and the flame, and that catches fire quickly [1]. As Rousell points out, a common feature of punk is its organic origin. This makes its conservation very rare, although the remains of some fruiting bodies have been found, most frequently as tinder preserved at waterlogged sites.

In order to be used as tinder, or more precisely, to maximize its efficacy, the fibres of the bracket fungi must be prepared. A previous preparation of the fungi improves their qualities as tinder. Nevertheless, this preparation has not always been identified in the archaeological remains. Some, like the ones of the above-mentioned Ötzi or the one found at Edlingen [12], display this previous preparation, but others, like the finds from La Draga, do not. In the case of fungi at La Draga, most of the finds consist of whole individuals or fragments without further preparation. It is obvious that the fragments that have reached us were not burned, so they may have been stored for future use or were discarded.

## Conclusions

Fungi, like other organic materials, are rarely recovered at archaeological sites due to their perishable nature. The exceptional preservation of organic materials at La Draga due to the waterlogged conditions has allowed the recovery of a fungal assemblage formed by seven taxa. All the taxa identified correspond to polypore fungi. According to their current hosts, all of them could have grown in the forest near the settlement. The relative abundance of remains, their ecology and characteristics suggest intentional transportation to the settlement. The most abundant (*Ganoderma adspersum*) is a parasite of living trees and therefore cannot have grown on the wooden structures at the settlement, located on the lake shore. In addition, fungi belonging to different ecosystems (deciduous and riparian forests) are represented in the same place, which reinforces the hypothesis of intentional transportation to the settlement. Finally, evidence of manipulation has been identified in two of them.

Remains of the same or similar polypore species have been recovered at other lake-shore sites across Europe. The fungal fruiting bodies identified at the La Draga site are not edible. Therefore, the interpretation based on current and ethnographical known uses seems to indicate a use as tinder, which would be perfectly coherent with the known fire technology used at the site.

## Supporting information

S1 Table Detailed list of analysed fungal remains. (XLSX)
